# BK Channelopathies and *KCNMA1*-Linked Disease Models

**DOI:** 10.1146/annurev-physiol-030323-042845

**Published:** 2023-10-31

**Authors:** Andrea L. Meredith

**Affiliations:** Department of Physiology, University of Maryland School of Medicine, Baltimore, Maryland, USA

**Keywords:** large-conductance calcium-activated potassium channels, potassium channels, channelopathies, movement disorders/dyskinesias, epilepsy, nervous system diseases, rare diseases, animal models

## Abstract

Novel *KCNMA1* variants, encoding the BK K^+^ channel, are associated with a debilitating dyskinesia and epilepsy syndrome. Neurodevelopmental delay, cognitive disability, and brain and structural malformations are also diagnosed at lower incidence. More than half of affected individuals present with a rare negative episodic motor disorder, paroxysmal nonkinesigenic dyskinesia (PNKD3). The mechanistic relationship of PNKD3 to epilepsy and the broader spectrum of *KCNMA1*-associated symptomology is unknown. This review summarizes patient-associated *KCNMA1* variants within the BK channel structure, functional classifications, genotype-phenotype associations, disease models, and treatment. Patient and transgenic animal data suggest delineation of gain-of-function (GOF) and loss-of-function *KCNMA1* neurogenetic disease, validating two heterozygous alleles encoding GOF BK channels (D434G and N999S) as causing seizure and PNKD3. This discovery led to a variant-defined therapeutic approach for PNKD3, providing initial insight into the neurological basis. A comprehensive clinical definition of monogenic *KCNMA1*-linked disease and the neuronal mechanisms currently remain priorities for continued investigation.

## INTRODUCTION

Potassium channelopathies are a class of rare monogenic diseases caused by mutations in K^+^ channel subunits (1). K^+^ channels form as homo- or heterotetramers from 80 KCN gene products within five structurally related families encoded in the human genome. Their activity contributes subthreshold current to regulate resting membrane potential and excitability set points, repolarizing current during action potentials, hyperpolarizing current after action potentials, and K^+^ flux across membranes. Thirty-five years after the first voltage-gated (K_V_) gene *Shaker* was cloned in flies, human K^+^ channel mutations have been shown to cause epilepsy (K_V_1, K_V_2, K_V_3, K_V_4, K_V_7, K_V_10,K_Ca_1,K_V_4, K_ir_6, K_Na_), cardiac arrhythmia (K_V_1, K_V_4,K_V_11,K_ir_2,K_ir_3, K_ir_6), dyskinesia and periodic paralysis (K_V_1, K_V_3, K_V_4, K_Ca_ 1, K_ir_2, K_ir_4), deafness (K_V_6, K_V_7, K_ir_4, K_ir_5), visual deficits (K_V_8, K_ir_7), cognitive and psychiatric disorders (K_V_4, K_V_7, K_V_10, K_V_11, K_Ca_1, K_Ca_2, K_ir_2, K_ir_3, K_2P_), migraine (K_2P_), renal dysfunction (K_ir_1, K_ir_4, K_ir_5), diabetes (K_ir_4, K_ir_6), pulmonary hypertension (K_2P_), and anemia (K_Ca_3). K^+^ channelopathy-associated gene variants impact numerous K^+^ channel properties, affecting assembly, trafficking, localization, and gating. Although initially characterized through recessive disease, recent widespread genetic testing has expanded the potential genotypic basis for channelopathy-related symptomologies. These new associations often lack familial pedigrees, involve heterozygous and variant of uncertain significance (VUS) alleles, and present significant challenges for substantiating monogenic disease caused by individual K^+^ channel gene variants.

*KCNMA1*-linked disorders are recently characterized and defined by mutations in the human BK K^+^ channel (K_Ca_1.1). The disorder manifests as various combinations of epilepsy (54% of patients), movement disorder (59%), intellectual disability (27%), neurodevelopmental delay (20%), and brain (24%) and visceral (9%) malformations, with other heterogeneous symptoms (2–5) (Figure 1). The clinical definition of the disease and the structural and functional BK channel mechanisms are under investigation.

The first disease-associated *KCNMA1* variant was not identified until 2005 (5), despite two decades of investigating BK channels as potential pharmacological targets in glaucoma, stroke, asthma, hypertension, urinary incontinence, erectile dysfunction, intellectual disability, ataxia, and seizure (2, 6–10). The D434G mutation and its clinical associations [generalized epilepsy and paroxysmal dyskinesia (GEPD)] were not then known to be part of a larger, more heterogeneous but predominantly neurological disorder (2–4) (Figure 1). Yet to date, most of the 110 individuals known to us do not have a standardized clinical diagnosis or a treatment plan. The disorder remains principally defined by the presence of a neurological diagnosis in conjunction with a *KCNMA1* gene mutation, identified through genetic testing and encompassing mainly de novo, heterozygous VUS alleles. The rarity of patients and inherited transmissions, in combination with symptomatic variation, has hampered genotype-phenotype correlation and understanding the mechanistic basis of symptoms. In comparison to *KCNT1* channelopathies caused by defects in the related K_Na_ channel, which has approximately double the number of patients and a more severe epileptic phenotype (9), the *KCNMA1* disease definition is far less developed.

## BK CHANNELS: GENE TO PHYSIOLOGY

### *KCNMA1* Gene

*KCNMA1*-associated flight and locomotor phenotypes were first observed in *Slowpoke* flies, which harbored a mutation in the gene locus encoding a Big K^+^ (BK) channel (11, 12). In humans, the BK channel α subunit encoded by *KCNMA1* comprises 45 exons spanning 753 kb on the complement strand of human chromosome 10q22.3 (Gene ID: 3778; https://www.ncbi.nlm.nih.gov/gene/3778). The first exon contains three potential start codons (13). Sixty-two human GenBank transcripts encode messenger RNAs (mRNAs) of ~4.4–14 kb (coding sequence ~3.6–3.9 kb), produced by alternative splicing at four main sites to include up to 11 alternative exons. Two splices involve a single alternative exon (sites 1 and 3). The well-characterized STREX (site 2) can include up to four combinatorial alternative exons, and the C-terminal site contains one of three alternative exons (14–16). Alternative splicing occurs in a tissue and developmentally regulated manner. One instance of an intron retention with functional consequences on BK channel properties has been identified in mouse (17).

### *KCNMA1* Expression

BK channels widely regulate excitability, calcium (Ca^2+^)-mediated signaling, and K^+^ efflux in species from worms to mammals. *KCNMA1* expression is found predominantly in excitable cells such as neurons and muscle, but also in nonexcitable salivary, bone, and kidney cells. Human transcript and protein data sets show consistently high expression in brain, smooth muscle, and reproductive tissues (Supplemental Figure 1). Brain expression is highest in cortex, hippocampus, amygdala, striatum, thalamus, and cerebellum. Hypothalamus, brain stem, spinal cord, peripheral nervous system, retina, and pituitary also exhibit notable *KCNMA1* or BK channel expression. Excitatory neurons generally show higher levels than inhibitory neurons. Neurons generally express more *KCNMA1* or BK channel protein than glia, and developed tissues express more than fetal tissues. Translational control of BK channel protein is regulated in some neurons by fragile *X* mental retardation protein (FMRP), now appreciated as a broad regulator of ion channel expression and function (18). The resolution of *KCNMA1* expression is limited by a lack of systematic expression studies in healthy human tissues. Where both have been assessed, transcript and protein levels are not always correlated. *KCNMA1* is expressed in a variety of laboratory cell lines and in some cancer cell lines, including brain, kidney, and prostate.

### BK Channel Structure

BK channels possess several distinguishing features compared to other K_V_ family members, including a significantly larger channel conductance with distinct pharmacology, dual regulation by an integral of voltage and intracellular Ca^2+^, and a pore-forming tetramer encoded by a single gene product (10, 13). Like K_V_ channels, each *KCNMA1*-encoded α subunit contains six transmembrane segments that form the membrane-spanning channel core, composed of the voltage-sensing domain (VSD; S1–S4) and a highly K^+^-selective pore (PD; S5–S6) (19) (Figure 2a,b). Differing from K_V_ channels, BK channels have an additional transmembrane segment (S0) that creates an extracellular N terminus involved in accessory subunit interactions (20, 21), a weaker voltage-dependent VSD (22, 23), and a large intracellular gating ring that binds divalent cations (24–26). The gating ring is formed by two regulator of conductance of K^+^ (RCK) domains covalently attached to the core via the S6 linker. Ca^2+^ binds at one high-affinity site within each RCK domain (8 Ca^2+^ per tetrameric channel), and a single low-affinity Mg^2+^ site is formed between residues in the S0–S1 and S4–S5 linkers and RCK1 (4 Mg^2+^ per channel).

BK channel opening is regulated by voltage and Ca^2+^ through concerted movements within the core and gating ring, described by allosteric models (10, 22, 27). In the closed state, residues within the βA-αC (AC) region at the N-lobe of RCK1 mediate a noncovalent interaction between the gating ring, S6 linker, and S6 domain, stabilizing the closed channel conformation. This interaction changes during activation. When Ca^2+^ binds at RCK1 and RCK2, the gating ring expands like “petals opening on a flower” (19, p. 94), altering the stability of the interaction through multiple pathways to promote pore opening (27). With voltage, the VSDs move to stabilize the open pore via a gating lever bridge between the VSD and S6. One of the consequences of this multipathway allosteric gating scheme is the ability for BK channels to operate under a wide range of physiological conditions, where the amount of each stimulus may vary. In most cell types, BK channel activity responds to dynamic depolarizing voltage across the membrane (dV/dt) and intracellular-free Ca^2+^ within nanodomains (dCa^2+^/dt) that varies from nanomolar to micromolar concentrations (28–32).

### BK Current Properties

BK channels are tailored for distinct functions across diverse tissues by a suite of variations in gating properties and interactions with other ion channels. Intrinsic BK channel properties are set by cell-specific alternative splicing, accessory subunits, subcellular localization, and intracellular signaling factors (Figure 3a). Alternative exons affect multiple aspects of voltage, Ca^2+^, and kinetic gating properties, depending on the exon combinations at four main sites (16): (*a*) RCK1 (33), (*b*) RCK1–RCK2 linker (STREX) (14), (*c*) before the Ca^2+^ bowl in RCK2 (34), and (*d*) the terminal exon (35). Gating properties are further regulated by association with nonobligatory beta and gamma subunits (β1–4, γ1–5), posttranslational modifications (such as ~30 phosphorylated serine/threonine residues identified in brain), and intracellular mediators (kinases, phosphatases, FMRP, redox state, protons, lipids, estrogen and steroid hormones, carbon monoxide, heme, and others) (10, 36–38). Many of these mechanisms are contingent, such as phosphorylation of STREX variants, or contextual, such as the magnitude and direction of shifts in activation produced by the β4 subunit in different Ca^2+^ conditions.

Extrinsic regulation of BK channel gating and properties occurs through depolarizing stimuli and changes in intracellular-free Ca^2+^ levels. Neuronal BK currents activate at depolarizations above −50 mV (39, 40), usually achieved through BK-Ca^2+^ channel coupling. Specific partnerships with voltage-gated Ca^2+^ channels (Ca_V_), Ca^2+^-permeable receptors, and store-operated Ca^2+^ release channels connect BK channel activation to highly localized subcellular Ca^2+^ dynamics (28, 41, 42). By providing hyperpolarizing feedback on Ca^2+^ channel activation, BK channels act as the brakes on excitability and Ca^2+^-mediated overexcitability in some contexts. However, as the number of recordings in different cell types has increased, it has become clear that BK channels shape membrane signaling under a variety of states (41) and require coupling with multiple types of voltage-gated and intracellular Ca^2+^ channels to accomplish this (30, 31, 40). Within these partnerships, BK currents activate and deactivate more slowly than voltage-gated and intracellular Ca^2+^ channels, imparting their voltage-dependent and kinetic influences on BK channel activation (32, 39, 43). Thus, BK and Ca^2+^ channel proximity has critical biophysical implications, as some of the physiological and pathophysiological mechanisms affecting BK channel gating differ with Ca^2+^ concentration (42).

BK channels are activated under various combinations of intrinsic and extrinsic regulations in vivo. BK channel opening is promoted by depolarizing potentials generated by action potential–initiating inward voltage-gated sodium channel (Na_V_) current, neurotransmitter-gated currents (i.e., NMDA receptor–mediated), tight spatial coupling to Ca^2+^ channels that produce micromolar increases in nanodomain Ca^2+^ (such as Ca^2+^ sparks), inclusion of the STREX (site 2) or Ca^2+^ bowl (site 3) exons, protein kinase A phosphorylation, β1 and β4 subunits, and γ subunits (10, 15, 34, 37, 44). BK channel activation is reduced during repolarization, decreased Ca^2+^ influx and reduced Ca^2+^ channel coupling, inclusion of the SRKR (site 1) exon, STREX-dependent PKA phosphorylation, and β2- and β3-mediated inactivation (33, 42, 45). The net effect of these regulatory mechanisms is highly context and state dependent. For example, in circadian clock neurons of the hypothalamus, transcript levels, alternative splicing, phosphorylation, β subunits, and different Ca^2+^ channels regulate BK current properties in a time-dependent manner to generate a daily oscillation in neuronal firing frequency (Figure 3b).

### BK Current Regulation of Neurophysiology

In mammalian neurons, BK currents are activated during action potential repolarization and the fast afterhyperpolarization (AHP), setting the frequency and pattern of firing. Pharmacological inhibition or genetic deletion of BK channels leads to an assortment of defects related to hyperexcitability (2, 41), typically presenting as neuronal action potentials with more rapid repolarization or reduced AHPs in the absence of hyperpolarizing BK current (Figure 3c). However, some cerebellar, vestibular, hippocampal, cortical, neurosecretory, and sinoatrial node cells paradoxically show decreased excitability upon BK channel inhibition (17, 46–53).

Whether BK currents cause this typically suppressive effect on firing by increasing the AHP, or whether they facilitate firing via speeding repolarization, depends on BK channel gating properties (46, 54, 55), their voltage-gated Ca^2+^ sources (43, 56, 57), and interplay with other currents (39, 58). In neurons, the β4 subunit is a primary modulator of activation, increasing open probability while simultaneously slowing activation. This has the net effect shifting BK current contribution from repolarization to the AHP but also depends on the Ca^2+^ source and the presence of other repolarizing K^+^ currents such as K_V_3 (39, 50, 56, 59, 60). Loss of β4’s slowing effect on gating increases neuron firing and produces seizures in mice (58), suggesting that fast-activating BK currents promote excitability. There is evidence for both fast- and slow-gating BK channels in hippocampal and cerebellar neurons (61), both areas of neuronal dysfunction in *KCNMA1* transgenic models (62–64). The β2 subunit also slows gating, but additionally bestows inactivation, conferring a multifaceted influence on neuronal excitability that is context-dependent. Loss of β2 results in burst firing under some conditions but decreases firing under others (45, 65). In nearly every context where they have been identified, the intrinsic and extrinsic gating factors creating these bidirectional influences of BK currents on neuronal excitability remain to be fully understood.

BK channel activation also regulates baseline membrane potentials under some conditions, affecting the balance of silent to actively firing cells (45, 65, 66), membrane oscillations (67, 68), and neurotransmitter release (69–72). Subthreshold BK activation in cochlear, chromaffin, and suprachiasmatic nucleus cells depends in part on low-voltage-activated Ca_V_1.3 channels and is modulated by the β2 subunit in the latter two cell types (40, 65, 68, 73). In smooth muscle, BK channels regulate contractile tone via a direct interplay with intracellular Ca^2+^ channels (44). Smooth muscle-specific β1 subunits increase the Ca^2+^ sensitivity of BK channels, promoting activation by ryanodine receptor–mediated Ca^2+^ sparks. This mechanism is present in vascular, bladder, uterine, and other smooth muscle tissues that highly express BK channels (44, 74, 75).

Many of these roles for BK channels in neuronal and muscle excitability are evolutionarily conserved from invertebrates to mammals and are altered by numerous engineered gain-of-function (GOF) and loss-of-function (LOF) channel mutations and patient variants (Figure 4). Notably, *Slowpoke* presaged BK channelopathy-related neuromuscular dysfunction in flies, manifesting as so-called sticky feet behavior that was explicitly dependent upon neuronal BK channel expression (12, 76).

## *KCNMA1* VARIANTS AND BK CHANNELOPATHIES

### Variant Identification

*KCNMA1* mutations are identified via genetic testing on 75 clinical panels for epilepsy, dyskinesia and dystonia, intellectual disability, and malformations (https://www.ncbi.nlm.nih.gov/gtr/all/tests/?term=3778[geneid]), or as rare variants from whole exome/genome sequencing. At present, most variants with curated patient symptomology localize within constitutive exons. Two variants occur in intronic regions, with single instances of a microdeletion and translocation (A. Meredith, unpublished data).

Prior to comprehensive genome annotations, the *KCNMA1* gene locus was independently implicated in a family of 13 affected individuals exhibiting GEPD via segregation analysis and positional cloning (5). This pedigree confirmed autosomal dominant inheritance, but >98% of affected individuals harbor de novo heterozygous *KCNMA1* variants. In these cases, dominance has been presumed based on the presence of neurological symptoms in heterozygotes. Only two patients harbor homozygous variants with the potential for recessive disease (77, 78). In most cases, direct evidence that the identified variants cause patient symptomology is lacking. Statistically validated *KCNMA1* variant-phenotype correlations within larger patient cohorts, or replication of symptomology in disease models generated from individual variants, are necessary to define pathogenic alleles in a monogenic disease scenario. Such correlation predicts that the variant would alter BK channel function (gating, localization, and/or interactions), alter BK current properties and firing in neurons and circuits associated with clinical dysfunction, and produce phenotypes similar to the core symptomology (i.e., seizure susceptibility and PNKD3) (62). In the absence of conclusive evidence for each individual variant, it is not possible to formally rule out whether *KCNMA1* is a polygenic risk contributor or even just an incidental finding not associated with disease pathogenesis.

### Location and Effect of Patient-Associated Variants Within the BK Channel

About one-third of the patient-associated *KCNMA1* variants have been studied for their effects on BK channel function (Figure 2c and Supplemental Table 1). Twenty-eight percent are determined to be LOF mutations, causing a decrease in BK current compared to wild-type (WT) by shifting activation to more depolarized potentials, slowing opening, speeding closing, and/or reducing expression. Four percent are GOF, increasing BK current by shifting activation to hyperpolarized potentials, speeding opening, and/or slowing closing. Sixty-eight percent have no functional designation (VUS), with nine of those reported benign under study conditions and the remainder functionally uncharacterized.

Thirty variants are located within the transmembrane channel core (Figure 2a,b). The majority of these are found in the first transmembrane segment and S0–S1 linker. Several deletions or duplications are found within a poly-glycine and serine-rich stretch on the extracellular N terminus preceding S0, a region that contains multiple initiator methionine residues and a high occurrence of single nucleotide polymorphism (SNP) variation (79). Two variants localize within S0, a region where mutations have the potential to affect the β subunit and Ca^2+^ channel interactions (20, 21). Of the six variants in the S0–S1 linker, three cause LOF gating: A138V, E155Q, and A172T (80–83). This loop contains one of the residues involved in Mg^2+^ coordination through interaction with the RCK1 N-lobe (26). A172T affects Mg^2+^-dependent gating (82), but A138V remains grossly Mg^2+^ sensitive (80). Variants located in this region may also affect trafficking, assembly, and interactions between the BK channel core and accessory subunits.

Only four variants occur within S1–S4 in the VSD region. One of these is N237K, presumed as LOF because it mutates a residue involved in Mg^2+^ binding located in the S2–S3 linker (26). Similar mutations (R or D) disrupt the electrostatic interaction, resulting in LOF (84). No variants are currently known to occur in any the charged residues directly involved in voltage sensing, although LOF A172T (S1) is adjacent to gating lever residues (27, 82). VUS T176S (S1) and L271F (S4) are also located near voltage-modulated residues (79).

The S5–S6 conduction pathway, where dehydrated K^+^ transits the membrane upon pore opening, contains seven patient variants. A314T (LOF; S5) is located in a region that could be relevant for VSD movement (27, 82). A cluster of variants localize at the selectivity filter (LOF: S351Y, G352A, G354S, G356R, G375R; VUS: T352A), near the tetraethylammonium, iberiotoxin, and charybdotoxin binding sites (4, 64, 85, 86). G354S and G356R mutate glycines of the conserved GYG signature sequence responsible for high K^+^ selectivity. G354S reduces the single channel conductance and alters ionic selectivity (64). G375R also reduces conductance (85). Unexpectedly, both G354S and G375R shift the voltage dependence in a GOF manner. Additional residues beyond the selectivity filter that concentrate K^+^ in the intracellular vestibule of the permeation pathway, generating the high conductance characteristic of BK channels (87), have not yet been mutated by patient-associated variants.

Most variants localize within the intracellular gating ring, which mediates Ca^2+^ binding and allosteric translation to pore gating (24, 25, 88–91). D434G, the first *KCNMA1* disease-associated mutation identified (5), is the mechanistically most well-studied variant with multilab corroboration. It is located in the AC domain near the RCK1 Ca^2+^ -binding site. D434G primarily affects Ca^2+^ sensitivity, enhancing allosteric coupling of gating ring movement by increasing AC region flexibility (92, 93). The AC region also contains three additional LOF variants (C413Y, H444Q, K457E) (4, 62, 94), two putative truncations (N449fs, R458X) (4, 95), and a cluster of sites involved in CO, H^+^, and ethanol modulation (79). K457 interacts with phosphatidylinositol 4,5-bisphosphate (PIP2) to promote BK channel activation and may be involved in β subunit modulation (96, 97). M578T (LOF) (79) and T603A (VUS) are near residues in RCK1 involved in Ca^2+^ modulation but are not within binding sites. The GOF variant N536H is located at the C terminus of RCK1, at the interface between adjacent α subunits, and shifts the voltage relationship without significantly affecting on Ca^2+^ sensitivity (98). Three variants are found in the linker between RCK1 and RCK2, near the site of STREX alternative splicing (site 2). One of these (D800Y) was reported benign under initial testing conditions (79).

RCK2, containing the Ca^2+^ bowl site, includes 22 patient variants. However, less is known about their functional effects. Two variants located near, but not within, the Ca^2+^ -binding site mutate charged aspartates (D984N and D965V) and create LOF properties (4, 79). Additional LOF variants R800W, P805L, and R1097H localize near each other at the C-terminal side of RCK2 (4, 80, 86). N999S (also called N995S; Supplemental Table 1) is another variant with multilab corroboration studying its GOF effects (62, 82, 99, 100). N999S is situated between these two LOF groups at the mid-S10 helix bend, 10 Å from D434G. Mechanistic studies introducing N999S with Ca^2+^ -binding site mutations suggest its effects are exerted predominantly through modulation of voltage-dependent gating (100). However, due to its distal location from the VSD, further analyses of the N999S GOF mechanism have the potential to reveal new RCK2-VSD connections during gating (27). Interestingly, despite the common phenotypes among patients with GOF variants in RCK1 (D434G and N536H) and RCK2 (N999S), no structural associations have been identified between these residues.

Besides gating alterations, seven variants decrease expression (A314T, S351Y, G356R, G375R, N449fs, N536H, I663V) (4, 82), and nine create frameshifts or premature terminations with the potential to affect channel expression or assembly (K124fs, L429fs, N449fs, R458X, K480fs, R830X, H841fs, R880X, N929fs). Functional data support LOF designations for N449fs, R458X, and Y676fs (4, 77, 78, 83). However, because patients are heterozygous, the potential to form WT/mutant heterotetramers of unknown properties exists. For example, truncated homotetramaric channels missing the gating ring can activate with voltage alone in the absence of Ca^2+^-binding sites. However, truncated channels show reduced coupling between the voltage sensors and the pore (101). In full-length BK channels (79, 102, 103), VSD-pore coupling is translated through the AC domain near R458X. R458X may produce different phenotypes under several recording conditions (83, 95). Therefore, functional classifications for variants affecting translation or expression will require advanced model systems that recapitulate patient allelic composition.

Recent studies addressing the heterozygosity issue showed that BK channels produced from equivalent amounts of coexpressed WT and G375R subunits were composed of 6% WT, 88% heterotetamer, and 6% mutant properties in oocytes (85), with a mixed LOF/GOF phenotype beyond that described in previous studies (4). Thus, the extent to which mutant BK channels recorded in heterologous cells (i.e., channels that are homotetramers of a single α isoform under clamped Ca^2+^ in symmetrical K^+^) accurately classify the activity of patient-variant channels in vivo merits continued experimental refinement. Better functional classifications would incorporate heterotetramers of alternately spliced α/β/γ-Ca^2+^ channel complexes under physiological ionic gradients and signaling conditions, within neurons directly implicated in the disease mechanism. Even so, experimental systems that incorporate BK channel complexes, endogenous regulatory mechanisms, and physiological stimuli may still need tailoring for particular *KCNMA1* variants. For example, within dentate granule neurons, BK channel LOF due to application of paxilline or the H444Q mutation has little effect, while the GOF mutation N999S causes hyperactive firing (58, 62) (Figure 3c).

### Genotype-Phenotype Associations: Gain-of-Function Channelopathy Variants

Of the 75 patient-associated *KCNMA1* variants, only three have been identified that produce entirely GOF BK channel activity (D434G, N536H, and N999S) (5, 98–100). Together these dominant GOF alleles comprise half of the known patient population (3). N999S is the most common, occurring as de novo heterozygous alleles in all individuals (3, 82, 100, 104–107). D434G (heterozygous) was followed through a multigeneration pedigree (5), and N536H (de novo heterozygous) was found in two individuals (82, 98). Based on evidence from the familial pedigree, statistical genotype-phenotype correlation, and transgenic animal models, D434G and N999S can be considered causative for epilepsy and dyskinesia (GEPD) (5, 62, 63). N536H is also presumed causative due to the similar phenotypic presentation (82, 98). Therefore, GOF BK channelopathies can be considered a monogenic *KCNMA1* disorder.

Most individuals harboring GOF BK channelopathies are diagnosed with episodic negative motor symptoms presenting as paroxysmal nonkinesigenic dyskinesia, non-narcoleptic cataplexy, hypotonia/atonia, and dystonia (Figure 1; see videos in 98, 107, 108). These episodes occur with relatively short duration and high frequency compared to PNKD linked to other genes, at tens to hundreds of attacks per day (109, 110). Affected individuals do not have motor or atonic seizures. Instead, the GOF-associated PNKD3 abates with stimulant therapy (82, 98, 104).

In addition, about half of the patients with GOF channelopathies are diagnosed with epilepsy (absence, atonic, myoclonic, generalized tonic-clonic, and epileptic encephalopathy) or have a childhood history of seizure (3). Although present in D434G mice (63), absence seizures have not been definitively implicated in GOF channelopathies by statistical correlation in human patients. Other symptoms including mild to moderate cognitive impairment and intellectual disability (autism spectrum disorder), and more recently, microcephaly and facial dysmorphisms (82), have also been identified in patients harboring GOF variants.

### Loss-of-Function Channelopathy Variants

More prevalent than GOF variants, individuals harboring putative LOF channelopathy variants that reduce BK channel activity in vitro also exhibit de novo, heterozygous alleles that are associated with an expanded range of impairments. LOF channelopathy variants are found in patients with epilepsy in similar proportions as the GOF cohort (3, 4, 77, 78, 82, 83). Most of these patients also have movement disorder (ataxia, dyskinesia, tremor, hypotonia, dystonia) (64, 78, 82, 83), but only a minority are diagnosed with PNKD3 (3, 62, 77, 106). Ataxia may be a common symptom of LOF channelopathies, supported by transgenic animal models for variants H444Q and G354S (62, 64). Intellectual disability and autism spectrum disorder (4, 77, 78, 80–82, 106), psychiatric disability (83), brain and structural malformations (4, 64, 77, 86), speech pathology (4, 64), neonatal diabetes (82, 86, 111), and gastrointestinal disorder (86) are additionally found in individuals harboring LOF channelopathy variants.

An LOF channelopathy syndrome has been proposed by the Liang Wang group (LIWAS; Figure 1) and is phenotypically defined by individuals harboring A172T, A314T, S351Y, G356R, G375R, C413Y, N449fs, I663V, P805L, and D984N variants (4, 82). These patients have dysmorphic features with cerebral atrophy and connective, cardiovascular, and bone polymalformations. Some patients also have epilepsy, developmental delay, cognitive impairment, ataxia, and hypotonia. Moreover, this group found LIWAS dysmorphic features in two individuals harboring N536H and N995S, variants previously not presenting with structural malformations in the larger patient cohort (82). However, unlike the case for GOF channelopathies, direct evidence establishing LIWAS (or any other LOF symptomatic subset) as a monogenic *KCNMA1* disorder from familial transmissions, genotype-phenotype statistical correlation, or transgenic animal models is not yet available.

## VARIANTS OF UNCERTAIN SIGNIFICANCE

Almost half of *KCNMA1* variants are classified as VUS at present, including 11 variants that are functionally uncharacterized but predicted pathogenic in silico (79) or benign under initial testing conditions (9 variants) (Figure 2c). Like GOF and LOF channelopathy variants, VUS symptomology spans a wide range, including seizure, PNKD and other movement impairment, neurodevelopmental and intellectual disability, psychiatric disability, and polymicrogyria and structural malformations (3, 86, 99, 100, 106, 112–115). Given the overall similarities in the VUS patient phenotypes compared to GOF and LOF channelopathy variants (3), it is likely that some (including putative benign variants) could be found to have deleterious BK channel or neuronal consequences under further testing. Nevertheless, the benign designation of at least one VUS does appear to be supported based on both BK channel and patient phenotype data. R1128W is an inherited heterozygous variant found in multiple individuals. It has no apparent pathophysiology in a proband carrying only R1128W and produces no additional effect on BK channel properties or patient symptoms in combination with N999S (99, 107, 115).

### Disease Models

By itself, *KCNMA1* genotype and BK channel phenotype correlation is not sufficient to unambiguously validate disease causation for individual variants. One reason is patient selection bias, where it is mainly individuals with intractable symptoms that are referred for genetic testing; moreover, diagnostic precision can vary based on the expertise of the primary specialty clinic. Whether the genetic testing was sufficient to identify ancillary findings (e.g., single- or multigene panels versus whole-exome/-genome sequencing methodologies) and whether the functional designations (LOF or GOF) accurately reflect variant-induced changes in BK channel activity under an array of physiological conditions are also factors. Additionally, most *KCNMA1* genotype-phenotype studies are underpowered, potentially contributing to a high false-positive association. With these caveats, animal models provide essential evidence addressing gaps in disease definition, specific gene variant-phenotype causations, and drug discovery.

Several animal models recapitulate *KCNMA1* GOF BK channelopathy phenotypes, increasing neuronal activity, seizures, and dyskinesia. All three patient variants creating GOF BK channels (D434G, N536H, and N999S) shift BK channel activation to more hyperpolarized potentials, with faster activation and slower deactivation (93, 98–100). This results in increased action potential–evoked currents in D434G- and N999S-expressing heterologous cells and transgenic neuron models (42, 62, 92, 99) (Figure 3c). CRISPR-based introduction of the most common patient variant (N999S) into the mouse *Kcnma1* gene increases BK current in heterozygous animals, confirming the mutant allele as hypermorphic in a setting replicating the patient genotypes. *Kcnma1*^N999S/WT^ mice show increased dentate gyrus neuronal firing and seizure susceptibility, as well as episodic hypokinetic dyskinesia similar to human PNKD3 (62). *Kcnma1*^N999S/WT^ mice have additional locomotor abnormalities including reduced motor coordination and strength. Like patients harboring the N999S variant (104), the PNKD-like immobility episodes in *Kcnma1*^N999S/WT^ mice are mitigated by amphetamine treatment (62).

GOF D434G channels have been more extensively studied, introduced into the mouse genome by several independent methods (62, 63, 116), and mutated at an orthologous residue in flies (117). Under equivalent conditions, D434G is a weaker GOF mutation compared to N999S at the BK channel level (99) and in mice (62). Yet together, the D434G and N999S transgenic models reveal that the basic phenotypes associated with GOF BK channel activity are neuronal hyperexcitability, increased seizure susceptibility, and dyskinesia. D434G is associated with increased cortical, dentate gyrus, and cerebellar neuronal activity (62, 63). These studies corroborate that GOF BK channelopathy, though mainly comprised of de novo *KCNMA1* variants and presenting with variable penetrance in humans, is a dominant monogenic disorder that can be replicated in flies, mice, and humans.

Detailed comparisons reveal some differences between N999S and D434G phenotypes. N999S is homozygous lethal in mice, similar to other GOF manipulations in BK channel activity (46), while D434G is not (62, 63). The D434G seizure and dyskinesia phenotype is more severe in homozygous versus heterozygous mice, but unlike N999S, homozygosity is required to produce PNKD-like dyskinesia (62, 63). These phenotypic differences may stem from the distinct mechanistic bases for these mutations. D434G is located in the AC domain of RCK1 and affects primarily Ca^2+^ sensitivity (93). N999S is located in RCK2 and affects gating in the absence of Ca^2+^ binding (100).

There is also phenotypic variation between the three independently generated D434G transgenic mouse models. A CRISPR-D434G mutation does not show spontaneous seizures but does show lowered evoked seizure thresholds and PNKD-like immobility in the homozygous configuration (62). A floxed-D434G mouse line had spontaneous absence seizures in the heterozygous configuration, dystonic-type dyskinesia evoked by ethanol and oxytremorine and cerebellar morphological changes (63). A BAC-D434G line has generalized tonic-clonic seizures without baseline motor deficits, although these mice were not tested specifically for PNKD (116). These comparisons suggest that factors in addition to the primary D434G mutation, such as surrounding genetic sequences or environment, could influence the phenotype. Like the differences observed between mouse models, the affected family carrying the D434G channel variant showed variable penetrance. Some individuals had only PNKD, while others had epilepsy and PNKD (5).

Other nonpatient-derived GOF models created via engineered mutations in the BK α or accessory subunits were also previously studied. A transgenic mouse line expressing the R207Q VSD mutation revealed that these GOF BK channel properties (fast activation that is shifted to hyper-polarized potentials) are capable of producing both increases and decreases in neuronal activity, depending on the context (46) (Figure 3c). Additionally, fast-gating BK channels from neurons lacking the β4 subunit have similarities with *Kcnma1*^N999S^ and *Kcnma1*^D434G^ in dentate granule action potential recordings (54, 56, 58). Like the GOF patient variant models, *Kcnmb4*^−/−^ mice have epilepsy (58) but have not been tested for dyskinesia. However, unlike *KCNMA1*, no epileptic patients with *KCNMB4* variants have been reported yet. *KCNMA1* variants producing GOF channels in the context of β4 deficiency would be predicted to be more severe than either one alone, as the mutations would both act in the same direction. Another example, deletion of the β2 subunit, also has the potential to increase BK current activation by both speeding activation kinetics and eliminating inactivation. Neurons from *Kcnmb2*^−/−^ mice show changes in firing in both directions under different conditions (45, 65).

By comparison, LOF channelopathies are not well defined syndromically or conclusively established as a monogenic disorder. Established LOF mouse models are homozygous null gene inactivations (49, 74, 83, 118). The physiological dysfunction in these homozygous knockout mice is broader than patient phenotypes, encompassing neurological, locomotor, cardiovascular, reproductive, urological, renal, and neuroendocrine deficits (2). Their translational relevance to patient phenotypes is not fully explored. Although two homozygous *KCNMA1* patient variants have been reported, neither is known to be completely null for BK channel activity (77, 78, 83). LIWAS does not currently have an animal correlate, and no existing transgenic *KCNMA1* manipulation exhibits the polymalformation features. Lastly, the only transgenic LOF mouse line created from a patient variant (H444Q) fails to show increased seizure susceptibility or PNKD (62).

Yet synthesizing across LOF examples, a consistent phenotype shared by all homozygous *Kcnma1*^−/−^ models overlapping with patient symptomology is extensive locomotor dysfunction. However, in these models the LOF-associated motor deficits differ from GOF-associated dyskinesia. *Kcnma1*^N999S^ and *Kcnma1*^D434G^ exhibit hypokinetic PNKD, but *Kcnma1*^−/−^ and *Kcnma1*^H444Q^ are hyperkinetic under the same testing conditions (62). Additional LOF phenotypes include ataxia, tremor, decreased strength, and reduced locomotor coordination (49, 74, 119–122). Ataxia, tremor, and impaired coordination are also symptoms of Ryegrass Staggers, a toxicity syndrome of livestock (120) that further implicates BK channel inhibition with motor pathogenicity. LOF *Kcnma1*^H444Q^ and AAV-BK_G354S_ mice have reduced grip coordination and ataxia, respectively (62, 64). Thus, LOF disease-associated motor dysfunction is prominent in animal models.

Ryegrass Staggers is also associated with severe convulsive seizures, but there are conflicting data about seizures in transgenic LOF models. No epilepsy has been reported to date in two independently generated *Kcnma1*^−/−^ lines (49, 74), LOF patient variant models *Kcnma1*^H444Q^ and AAV-BK_G354S_ (62, 64), or *Slowpoke* flies (123). Conversely, a third *Kcnma1*^−/−^ mouse line is reported to have epilepsy (83), and *Slo* flies show increased susceptibility under conditions of alcohol withdrawal (124). This suggests the general potential for LOF variants to contribute to seizure, but paradoxically, BK channel inhibition with paxilline can also decrease seizure activity in chemo-convulsant models and D434G mice (63, 125). These contradictory findings may involve the observation that BK channel expression changes with epilepsy induction (126–128), creating the potential for dynamic summations of the BK channel expression and biophysical gating defects between animal models or different patient variants.

Assessments of other clinical symptomologies such as learning, memory, and morphological abnormalities have not yet been widely assessed in transgenic models harboring patient variants. However, AAV-BK_G354S_ (LOF) expression is associated with reduced neurite growth, viability, and mitochondrial function (64). The associated cerebellar phenotypes also fit with the broader implication of cerebellum in autism and learning disability. Similarly, rotarod performance in the GOF models *Kcnma1*^N999S^ and *Kcnma1*^D434G^ is suggestive of learning impairment, not just baseline motor dysfunction (62, 63). Furthermore, *Kcnma1*^N999S^ and *Kcnma1*^D434G^ mice also have decreased viability; however, the underlying mechanism is not yet known. Other nonpatient-derived models, such as homozygous *Kcnma1*^−/−^ knockouts, exhibit many neuronal, neurodevelopmental, and learning abnormalities and some osteoblast abnormalities (Figure 4). Moreover, there is growing evidence for BK channel dysfunction in the context of other animal models of learning disability (fragile X syndrome, autism spectrum disorder, and Angelman syndrome) and locomotor dysfunction (cerebellar and spinocerebellar ataxia) (2, 6, 7, 129–131). These studies suggest the possibility of uncovering additional phenotypes related to the core symptomology of patients in further channelopathy animal model investigations.

### BK Channelopathy Therapeutics

There are two aspects to BK channelopathy therapeutics. Initially, BK channels were worked up as novel pharmaceutical targets within a non-*KCNMA1* primary disease mechanism (2, 6–10), yielding an ocular glaucoma drug and several smooth-muscle targeted gene therapy pilot studies (132, 133). However, these treatments provide limited applicability in BK channelopathy. One long-standing additional complication has been the possibility that BK channel modulation in either direction could result in neuronal hyperexcitability and potentially contribute to epileptogenesis and motor dysfunction. The occurrence of seizure and locomotor disorder in both GOF and LOF channelopathy patient cohorts has not reduced this concern. Recently, the BK channel activator MaxiPost was shown to cause migraine in a randomized clinical trial (134). Thus, a better understanding of BK channelopathy mechanisms in patients and animal models is necessary to inform precision medicine strategies for new BK channel modulators.

The severe or refractory epilepsy and movement disorder experienced by the majority of patients are both areas of critical need for new drugs. In PNKD3, trigger avoidance (positive and negative valence emotions, tactile and temperature stimuli, fatigue, and alcohol) is necessary because the mechanisms for most paroxysmal dyskinesias are not well understood (109, 110, 135). Some paroxysmal dyskinesias respond to antiepileptic medications such as carbamazepine, but in GOF-associated PNKD3, amphetamine stimulants have been singularly effective in reducing the attacks in patients harboring N999S and N536H channel variants (98, 104, 108). For this variant-specific therapy, treatment considerations usually focus on dosing regimes to maximize the therapeutic window without detriment to sleep. The corrective effect is also corroborated in the *Kcnma1*^N999S^ mouse model, which responds to dextroamphetamine with a reduction in immobility during attacks (62). Neither amphetamine formulation prescribed to patients with PNKD3 (lisdexamfetamine or dextroamphetamine) appears to directly affect BK channel activity in heterologous cells (62, 98), suggesting that they work via a distinct target. BK channels are expressed in a variety of amphetamine-responsive and motor circuitry (cortex, striatum, cerebellum, neuromuscular junction), but no studies have identified the underlying neuronal pathophysiology in PNKD3 yet. Although presumed centrally generated, the contribution of other factors such as altered neuromuscular transmission or skeletal muscle hypotonia remains to be assessed during patient attacks.

A few additional drugs have been trialed in other types of LOF-associated movement disorder. The K_Ca_ channel activator chlorzoxazone partially improved cerebellar ataxia in a patient harboring the G354S channel variant (64). In the same study, the BK channel agonist NS1619 activated heterologously expressed BK_G354S_ channels and restored neurite outgrowth and viability in AAV-BK_G354S_-expressing PC12 cells, suggesting an additional neuronal benefit to normalizing BK channel activity. The K^+^ channel antagonist 3,4-diaminopyridine has also been used for status dystonicus in an individual harboring the K457E LOF channel variant (94), suggesting a broader set of mechanistic possibilities in the motor disease aspects of BK channelopathies. BK channel modulation has also been proposed for non-*KCNMA1*-associated movement disorder. In spinocerebellar ataxia models, pathophysiological BK channel alterations are mitigated with chlorzoxazone (136).

Unlike the progress with movement disorder, no consensus has emerged yet with respect to epilepsy and BK channelopathies, hampered by heterogeneity and a lack of *KCNMA1* variant-specific correlation (3). Traditional antiseizure medications are implemented in *KCNMA1*-associated case reports, with variable efficacy (2). There are no modulators directly targeting BK channels currently available in the clinic. Although the antiepileptic drug acetazolamide was reported to activate BK channels, suggesting a specific mechanism to mitigate LOF-associated seizures, the effect has not been substantiated in heterologously expressed BK channels (99). On the other hand, a few studies suggest that epilepsy could be one area where direct BK channel inhibition would be successful. Chemoconvulsant-evoked and BK-D434G epilepsy models both show a reduction in seizures with the BK channel inhibitor paxilline (63, 125, 137, 138). However, paxilline is not approved for human use, and BK channel inhibition strategies cannot currently be tested in patients.

Ca^2+^ channel inhibitors flunarizine, nimodipine, nifedipine, verapamil, ethosuximide, gabapentin, and dantrolene have been investigated as monotherapy or adjunct therapy in some types of epilepsy, movement disorder, and other neurological conditions (139–145). These approved modulators suggest an approach to reducing GOF BK channel activity through inhibition of the Ca^2+^ sources that activate neuronal BK channels. The antiepileptic T-type inhibitor ethosuximide suppressed seizure activity in BK-D434G mice (63) but has few reported treatments in patients with *KCNMA1* variants (2). Moreover, the antihypertensive voltage-gated Ca^2+^ channel inhibitor verapamil has been suggested to directly inhibit BK channels (146), but this has not been further substantiated. Because BK channels form complexes with Ca^2+^ channels in a neuron-specific manner, their inhibition has some potential for targeting specificity. However, potential cardiac side effects remain a barrier.

To address the gap in available therapeutics, progress toward developing new BK channel pharmacology and gene therapy is needed. No drugs currently exist with the potential to treat LOF channel variants that decrease expression. However, progress with structural analysis of patient-associated mutations may reveal new strategies for precision pharmacological targeting. The channel pore contains an obvious region where antagonists and LOF mutations overlap (79). The AC region and N-lobe of RCK1 have also emerged as regions of multimodal integration, harboring a cluster of patient mutations that overlap with sites of endogenous modulation (79) and residues involved voltage and Ca^2+^-domain coupling (27). Interestingly, despite the more homogeneous clinical presentation for GOF BK channel patient variants, their locations within the gating ring are disparate. New studies addressing GOF mechanistic commonalities may further improve our understanding of BK channel activation toward new directions for novel LOF-directed pharmacology.

## CONCLUDING REMARKS

*KCNMA1*-encoded BK channels widely regulate excitability across many physiological systems, but human mutations have revealed the predominant vulnerability of central neuronal functioning in patient-associated BK channel dysfunction. Moving beyond initial gene-symptomology correlation, transgenic models now validate the first *KCNMA1* variants that cause monogenic disease. Recent therapeutic advances create the possibility for decreasing symptomatic burden for a subset of patients with the most common GOF channelopathies. Collectively, BK channel functional alterations, disease models, and therapeutic responses provide the first inroads to elucidating the neuronal mechanisms, which may broadly benefit the understanding and treatment of other epileptic and movement disorders.

## Supplementary Material

supplemental

## Figures and Tables

**Figure 1:**
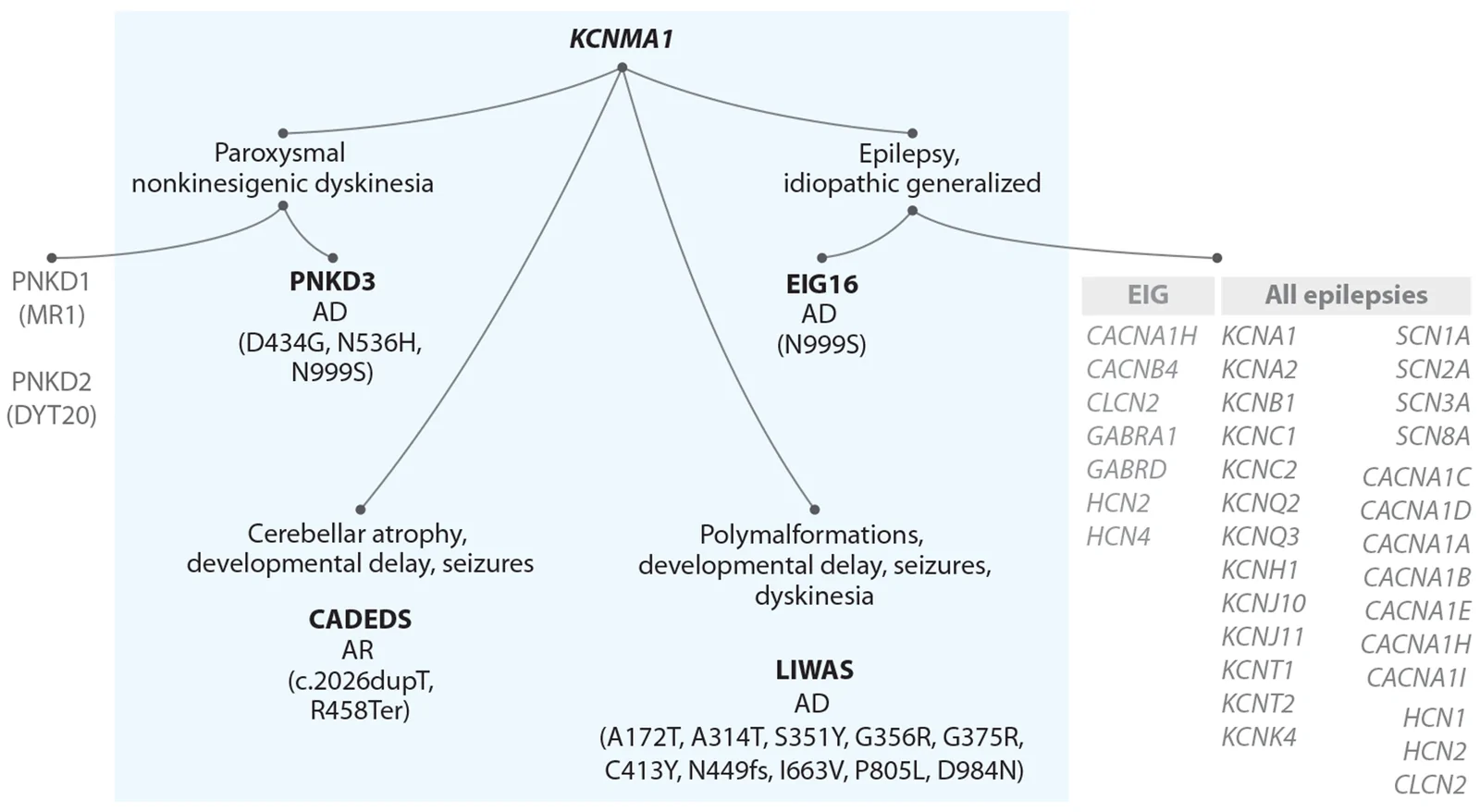
*KCNMA1* phenotypic dendrogram. The core diagnoses found among neurological patients with *KCNMA1* variants are movement disorder, epilepsy, intellectual disability, neurodevelopmental delay, and structural abnormalities. (*Blue box*) *KCNMA1* (MIM600150), PNKD3 (MIM609446), CADEDS (MIM617643), LIWAS (MIM618729), and EIG16 (MIM618596). Other MIM-identified epileptic channelopathies (EIG phenotypic series and all ion channels) and PNKD associations are shown outside the box. Data from Online Mendelian Inheritance in Man, OMIM^®^. Johns Hopkins University, Baltimore, MD. MIM Number: 600150: 01/07/2020. Accessed on December 15, 2022. https://omim.org/. Abbreviations: AD, autosomal dominant; AR, autosomal recessive; CADEDS, cerebellar atrophy, developmental delay, seizures; EIG, epilepsy-idiopathic generalized; LIWAS, Liang-Wang syndrome; MIM, Mendelian Inheritance in Man; PNKD, paroxysmal nonkinesigenic dyskinesia.

**Figure 2:**
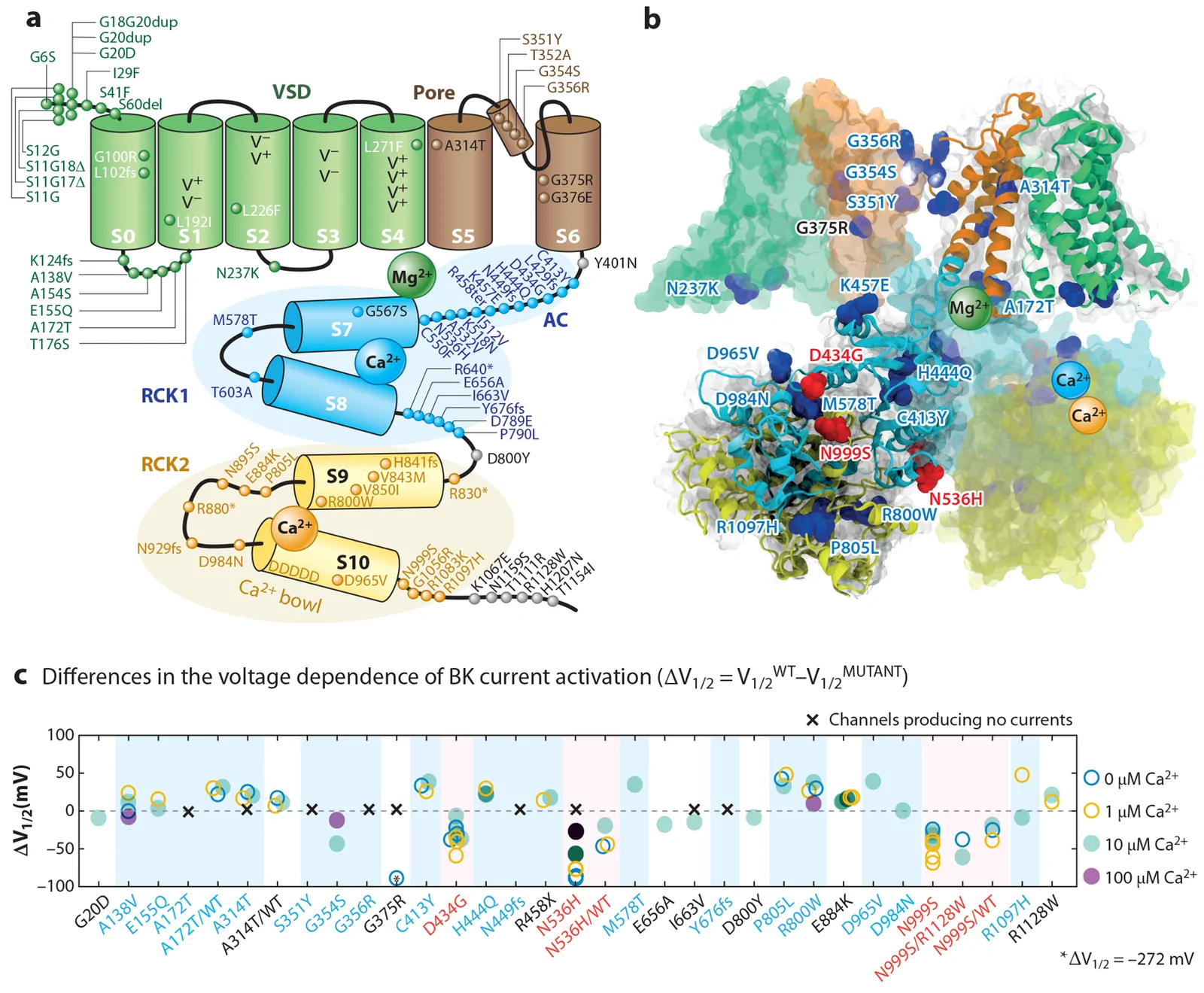
BK channel structure and *KCNMA1* variants. (*a*) BK channel transmembrane topology and intracellular gating ring (segments S0–S10) with 75 patient-associated *KCNMA1* variants. (*b*) Ca^2+^-bound cryo-electron microscopy structure (PDB ID: 6V38) (24) with GOF (*red*) and LOF (*blue*) variants. The VSD and voltage-modulated residues (V^+^ and V^−^), pore domain, βA-αC (AC) region, Mg^2+^-binding site, and two high-affinity Ca^2+^-binding sites located in RCK1 and RCK2 are depicted. (*c*) Functional effects of *KCNMA1* variants studied by BK channel electrophysiology or expression. Variants are numbered according to the original publications (accession numbers and citations for V_1/2_ values listed in Supplemental Table 1) and labeled by color: GOF (*red*), LOF (*blue*), and variant of uncertain significance or benign (*black*). Variants with data obtained from more than one condition or study are indicated by multiple symbols. Abbreviations: BK, Big K^+^; Ca^2+^, calcium; GOF, gain-of-function; K^+^, potassium; LOF, loss-of-function; PDB, Protein Data Bank; RCK, regulator of conductance of K^+^ domains; VSD, voltage-sensing domain.

**Figure 3:**
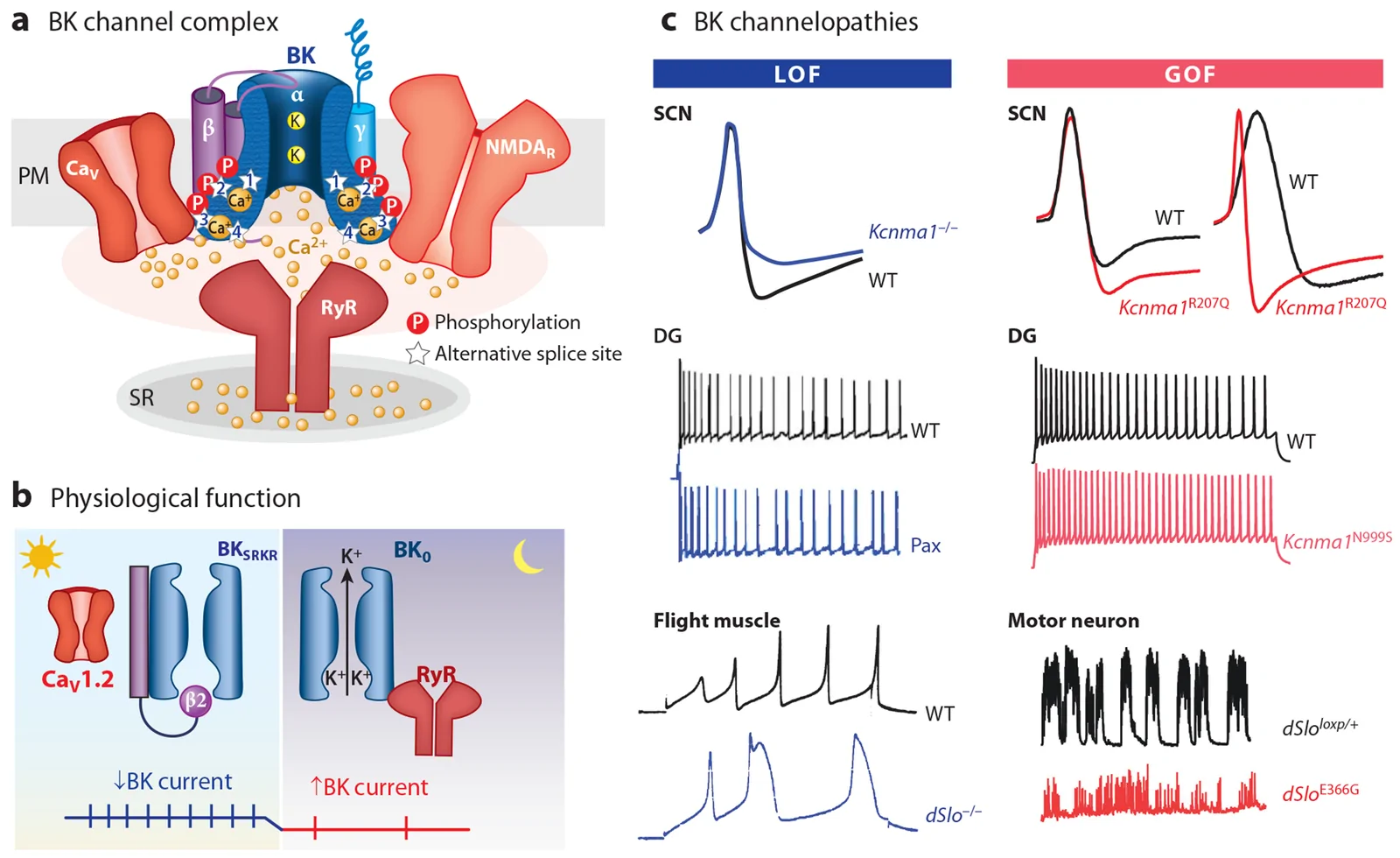
Physiology and pathophysiology of BK channels. (*a*) Alternative splicing (*star symbols*), phosphorylation, modulatory subunits (β1–4, γ1–5), and coupling to CaV channels, Ca^2+^ -permeable channels (NMDAR), and intracellular Ca^2+^ release channels (RyRs) regulate BK channel activation properties in a cell-specific manner. (*b*) State-dependent changes in SCN clock neuron excitability. Daytime BK current is decreased through BKSRKR isoform expression, S642 phosphorylation, β2-mediated inactivation, and Ca_V_ 1.2 channels, facilitating higher firing rates. Nighttime firing is suppressed by an increase in BK current, occurring through BK_0_ expression, decreased inactivation, and RyR channels. Disruption of each of these regulatory mechanisms alters action potential firing rate and time-of-day encoding (33, 40, 65, 66, 119). (*c*) LOF and GOF BK channel effects on action potentials from mouse SCN and DG neurons and *Drosophila* larval flight muscles (*left*) and motor neurons (*right*). Panel *c* adapted with permission from the following sources: left middle, Reference 58, copyright 2005 Springer Nature; left bottom, Reference 12, copyright 1986 NAS; right top, Reference 46, copyright 2012 NAS; right middle, Reference 62 (CC BY 4.0); right bottom, Reference 117 (CC BY 4.0). Abbreviations: β, beta subunit; BK, Big K^+^; Ca^2+^, calcium; Ca_V_, voltage-gated Ca^2+^ channel; DG, dentate gyrus; *dSlo*, *Drosophila* BK channel gene; γ, gamma subunit; GOF, gain-of-function; K^+^, potassium; LOF, loss-of-function; NMDA_R_, N-methyl-d-aspartate receptor; P, phosphorylation site; Pax, paxilline BK channel inhibitor; PM, plasma membrane; RCK, regulator of conductance of K^+^ domains; RyR, ryanodine receptor; SCN, suprachiasmatic nucleus; SR, sarcoplasmic reticulum; WT, wild-type.

**Figure 4:**
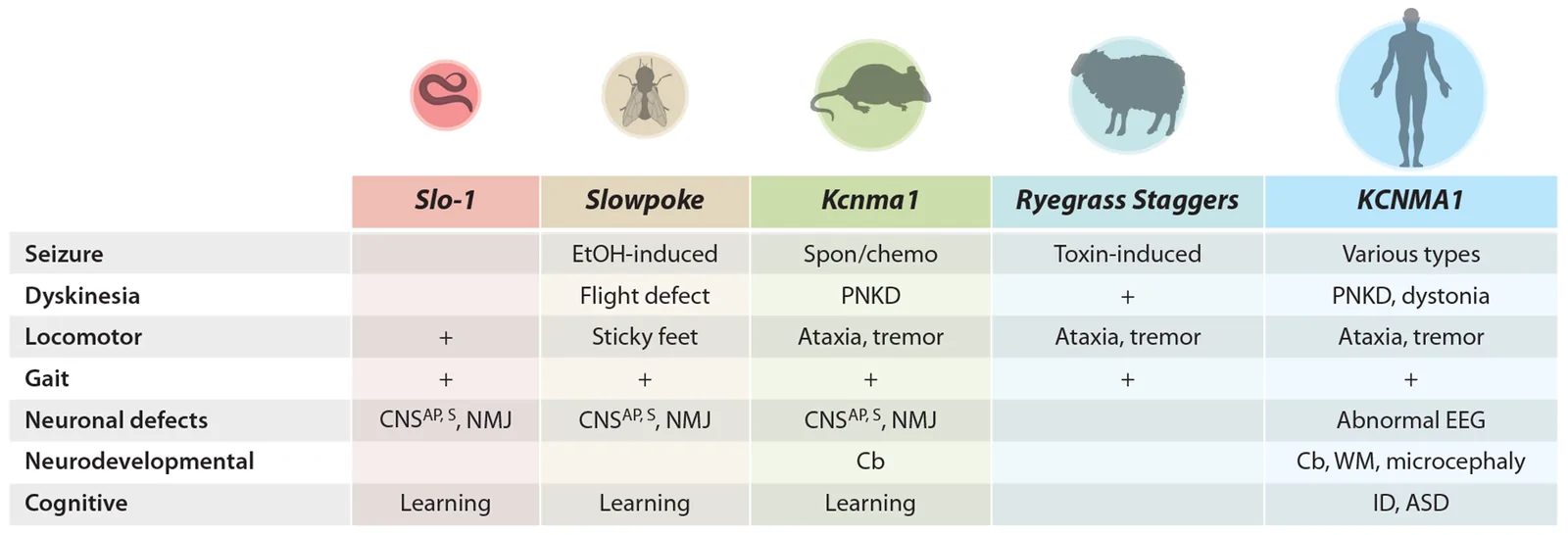
BK channel–associated neurological and locomotor dysfunction in transgenic models across species. Phenotypes associated with null, loss-of-function, and gain-of-function alleles. Worm: *Slo-1* and *Slo-1*(null);*hSlo1*(+) (147, 148). Fly: *Slowpoke* (11, 12, 149) and *Slo*^*E366G*^ (117). Mouse: *Kcnma1*^−/−^ (49, 83, 119); *Kcnma1*^*fl*^-*tdTomato* (118); Tg-BK^R207Q^ (46); *Kcnma1*^D434G^, *Kcnma1*^H444Q^, *Kcnma1*^N999S^ (62); *Kcnma1*^D434G (fl)^ (63); *Kcnma1*^D434G (BAC)^ (116); *Kcnmb1*^−/−^ (44); *Kcnmb2*^−/−^ (45); *Kcnmb4*^−/−^ (58); LRRC26^−/−^ (γ1) and LRRC52^−/−^ (γ2) (37). Sheep/cattle: *Ryegrass Staggers* (120). Human: 75 patient-associated *KCNMA1* variants (2–4, 79). Abbreviations: +, presence of symptoms; AP, action potential; ASD, autism spectrum disorder; BK, Big K^+^; Cb, cerebellum; chemo, chemoconvulsant-induced seizure; CNS, central nervous system; EEG, electroencephalogram; fl, floxed allele; ID, intellectual disability; NMJ, neuromuscular junction; PNKD, paroxysmal nonkinesigenic dyskinesia; S, synaptic; spon, spontaneous seizure; WM, white matter.

## SUMMARY POINTS

1. Patients harboring mutant gene variants in *KCNMA1* have been known for 20 years. The majority of patients recently identified harbor heterozygous, de novo variants.
2. Core symptomology for *KCNMA1*-associated disease includes epilepsy, movement disorder, neurodevelopmental abnormalities, intellectual disability, and brain and visceral malformations.
3. Generalized epilepsy and paroxysmal dyskinesia (GEPD) is the most consistent clinical phenotype within the symptomatic spectrum, associated with three gain-of-function (GOF) BK channel variants (D434G, N536H, and N999S).
4. Current successful therapies for GOF*-*associated epilepsy and dyskinesia do not directly target BK channel function.

## FUTURE ISSUES

1. Do patient-associated *KCNMA1* variants identify novel BK channel residues involved in allosteric gating?
2. Is heterologous BK channel expression an accurate predictor for *KCNMA1* variant pathogenicity in vivo? Can pathogenicity prediction be improved through primary functional investigations in induced pluripotent stem cell (iPSC)-derived neurons or transgenic animal models that recapitulate patient allele zygosity and physiological regulatory contexts?
3. Do all *KCNMA1* variants cause monogenic disease? Are there distinct GOF and loss-of-function (LOF) channelopathy disorders? With so few patients, will systematic and standardized clinical phenotype data collection (https://www.kciaf.org/registry) facilitate statistical genotype-phenotype correlations from pooled individuals?
4. Does refractory epilepsy in *KCNMA1* patients have a distinct mechanistic underpinning?
5. What are the locus and neuropathology of paroxysmal nonkinesigenic dyskinesia (PNKD3)? Is D434G-associated PNKD3 triggered differently from N999S-associated PNKD3? Is peripheral neuromuscular function also compromised during hypokinetic episodes?
6. What is the prognosis for BK channelopathies over the age span?
7. What is the success rate for traditional ion channel modulators, antiseizure, and antidyskinesia medications, and does the effectiveness differ between *KCNMA1* variants?
8. Are worm, fly, mouse, and human iPSC models suitable for pharmacological screening and evaluating efficacy and specificity?
