# Supplementary material for: BK Channelopathies and KCNMA1-Linked Disease Models
Source: Annu Rev Physiol. Author manuscript; Available in PMC 2026 Oct 16. (PMC13674577; doi:10.1146/annurev-physiol-030323-042845)
Supplement: Supplementary file 1 [file NIHMS2214165-supplement-supplemental.pdf]

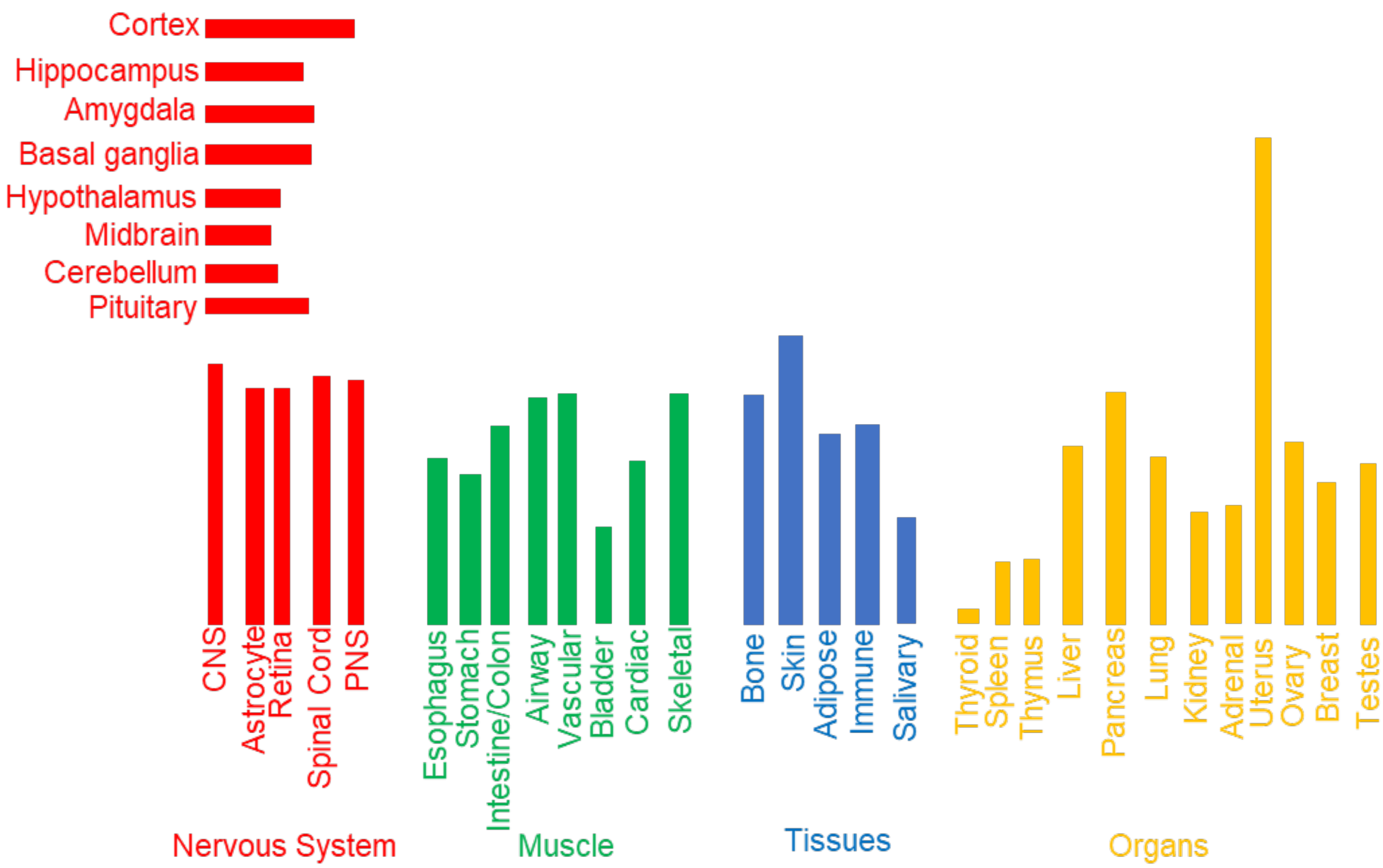

Supplemental Figure 1. ***KCNMA1* expression**
Transcript expression in normal human tissue combined from ARCHS[4] (https://maayanlab.cloud/archs4/gene/kcnma1), Gene (https://www.ncbi.nlm.nih.gov/gene/3778), and Human Protein Atlas (https://www.proteinatlas.org/ENSG00000156113-KCNMA1) databases (1-3). Levels from different databases were normalized to total brain for visualization.

Downloaded from www.annualreviews.org. Guest (guest) IP: 173.79.247.107 On: Thu, 01 Oct 2026 23:36:35

Downloaded from www.annualreviews.org. Guest (guest) IP: 173.79.247.107 On: Thu, 01 Oct 2026 23:36:35

Supplemental Table 1. ***KCNMA1*** **variant accession numbers and references**

Variant	Accession Number	Reference
G6S	VCV000532940.18	
S11G	VCV000300974.7	
S11G17del	rs1484259264	
S11G18del	rs587780364	
S12G	VCV000193223.23	
G18G20dup	rs760628050	
G20dup	VCV000193225.19	
G20D	rs888320237	(4)
I29F	rs746586408	
S41F	VCV000854906.5	
S60del	VCV000447640.10	
G100R	c.298G>C, pGly100Arg; NM_002247.3	
L102fs	VCV001072449	
K124fs	VCV000817829.2	
A138V	rs144215383	(5, 6)
A154S	VCV000501214.13	
E155Q	c.463G>C	(7)
A172T	VCV001047433.6	(8)
T176S	VCV001319205.1	
L192I	VCV001479179.4	
L226F	c.676C>T; p.Leu226Phe; NM_001161352.2	
N237K	VCV001312679.1	
L271F	VCV000945789.1	
A314T	c. 940G>A; p.A314T	(8)
S351Y	VCV000804292.1	(9)
T352A	VCV000216948.1	
G354S	rs1564596167	(10)
G356R	VCV000804293	(9)
G375R	rs1554829003	(9, 11-13)
G376E	VCV000983014.1	
Y401N	c.1201T>A; p.Tyr401Asn; NM_001322830.1	
C413Y	Het with N499fs; NM_002247.3	(9)
L429fs	VCV001414943	
D434G	VCV000009427	(4, 5, 8, 14-19)
H444Q	rs137853333	(4, 20)
N449fs	Het with C413Y; NM_002247.3	(9)
K457E	c1369A>G; p.Lys457Glu; NM_001161352.2	(21)
R458ter	VCV000691495	(7, 22)
I512V	rs781682363	(23)
K518N	rs770007121	(24)
A532V	VCV002433005.1	
N536H	c.1606A>C	(8, 25)

Downloaded from www.annualreviews.org. Guest (guest) IP: 173.79.247.107 On: Thu, 01 Oct 2026 23:36:35

C550F	c.1649G>T; p.Cys550Phe; NM_001161352.1	
G567S	VCV000981162.1	
M578T	c.1733 T>C; p.Met578Thr; NM_001161352.1	(4)
T603A	VCV001004014.5	
R640ter	rs1407652513	
E656A	rs149000684	(4, 24)
I663V	VCV000804294	(4, 9)
Y676fs	rs762705295	(26)
D789E	VCV000129322.10	
P790L	c.2369C>T; p.Pro790Leu; NM_001161352.1	(27)
D800Y	rs142210216	(4)
R830ter	VCV000942734	
H841fs	VCV001416721	
V843M	VCV001297508.3	
V850I	VCV000464295.15	
R800W	VCV001311186.3	(5)
P805L	rs150678882	(9, 11)
E884K	rs1554966197	(7, 28)
N895S	rs1565091862	
R880ter	rs2065361594	
N929fs	VCV000859287	
D984N	VCV000804295	(9)
D965V	c.2894 A>T; p.D965V; NM_002247.3	(4)
N999S (also called N995S, N1053S)	rs886039469	(4, 8, 20, 24, 28-32)
G1056R	c.3166 G>A; p.G1056R; NM002247.3	
R1083K	c.G3248A; p.R1083K; NM_001161352	(33)
R1097H	VCV002092623.1	(4)
K1067E	c.3199A>G; p.K1067E; NM_002247.3	
T1111R	Thr1111Arg; NM002247.3	
R1128W	rs747029218	(4, 16)
N1159S	rs563967757	(24)
T1154I	rs200773083	
H1207N	VCV001327422.1	

Patient-associated variants numbered according to the original publication (column 1), with Clinvar accession number, allele ID, or reference sequence (column 2). Unpublished de-identified data was collated under University of Maryland School of Medicine IRB NHSR designation HP-00083221, HP-00086440, and HP-00092434.

Supplemental References

1. Lachmann A, Torre D, Keenan AB, Jagodnik KM, Lee HJ, et al. 2018. Massive mining of publicly available RNA-seq data from human and mouse. *Nat Commun* 9:1366
2. NCBI. 2023. Gene [Internet]. Bethesda (MD): National Library of Medicine (US), National Center for Biotechnology Information; 2004 – [cited 2023 Jan 04]. Available from: https://www.ncbi.nlm.nih.gov/gene/3778
3. Sjostedt E, Zhong W, Fagerberg L, Karlsson M, Mitsios N, et al. 2020. An atlas of the protein-coding genes in the human, pig, and mouse brain. *Science* 367: eaay5947
4. Moldenhauer H, Tammen, K, Meredith, AL. 2023. Structural mapping of patient-associated KCNMA1 gene variants. *bioRxiv* https://doi.org/10.1101/2023.07.27.550850
5. Plante AE, Lai MH, Lu J, Meredith AL. 2019. Effects of Single Nucleotide Polymorphisms in Human *KCNMA1* on BK Current Properties. *Front Mol Neurosci* 12: 285
6. Laumonnier F, Roger S, Guerin P, Molinari F, M'Rad R, et al. 2006. Association of a functional deficit of the BK_{Ca} channel, a synaptic regulator of neuronal excitability, with autism and mental retardation. *Am J Psychiatry* 163: 1622-9
7. Yao Y, Qu D, Jing X, Jia Y, Zhong Q, et al. 2021. Molecular Mechanisms of Epileptic Encephalopathy Caused by *KCNMA1* Loss-of-Function Mutations. *Front Pharmacol* 12: 775328
8. Liang L, Liu H, Bartholdi D, van Haeringen A, Fernandez-Jaen A, et al. 2022. Identification and functional analysis of two new de novo *KCNMA1* variants associated with Liang-Wang syndrome. *Acta Physiol* 235(1): e13800
9. Liang L, Li X, Moutton S, Schrier Vergano SA, Cogne B, et al. 2019. *De novo* loss-of-function *KCNMA1* variants are associated with a new multiple malformation syndrome and a broad spectrum of developmental and neurological phenotypes. *Hum Mol Genet* 28:2937-51
10. Du X, Carvalho-de-Souza JL, Wei C, Carrasquel-Ursulaez W, Lorenzo Y, et al. 2020. Loss-of-function BK channel mutation causes impaired mitochondria and progressive cerebellar ataxia. *Proc Natl Acad Sci* 117: 6023-24
11. Rodrigues Bento J, Feben C, Kempers M, van Rij M, Woiski M, et al. 2021. Two novel presentations of *KCNMA1*-related pathology-Expanding the clinical phenotype of a rare channelopathy. *Mol Genet Genomic Med*: e1797
12. Mameli C, Cazzola R, Spaccini L, Calcaterra V, Macedoni M, et al. 2021. Neonatal Diabetes in Patients Affected by Liang-Wang Syndrome Carrying *KCNMA1* Variant p.(Gly375Arg) Suggest a Potential Role of Ca^{2+} and Voltage-Activated K^+ Channel Activity in Human Insulin Secretion. *Curr Issues Mol Biol* 43: 1036-42
13. Geng Y, Li P, Butler A, Wang B, Salkoff L, Magleby KL. 2023. BK channels of five different subunit combinations underlie the de novo *KCNMA1* G375R channelopathy. *J Gen Physiol* 155(5):e202213302
14. Wang B, Rothberg BS, Brenner R. 2009. Mechanism of increased BK channel activation from a channel mutation that causes epilepsy. *J Gen Physiol* 133: 283-94
15. Yang J, Krishnamoorthy G, Saxena A, Zhang G, Shi J, et al. 2010. An epilepsy/dyskinesia-associated mutation enhances BK channel activation by potentiating Ca^{2+} sensing. *Neuron* 66: 871-83

Downloaded from www.annualreviews.org. Guest (guest) IP: 173.79.247.107 On: Thu, 01 Oct 2026 23:36:35

Downloaded from www.annualreviews.org. Guest (guest) IP: 173.79.247.107 On: Thu, 01 Oct 2026 23:36:35

16. Moldenhauer HJ, Matychak KK, Meredith AL. 2020. Comparative gain-of-function effects of the *KCNMA1*-N999S mutation on human BK channel properties. *J Neurophysiol* 123: 560-70
17. Diez-Sampedro A, Silverman WR, Bautista JF, Richerson GB. 2006. Mechanism of increased open probability by a mutation of the BK channel. *J Neurophysiol* 96: 1507-16
18. Du W, Bautista JF, Yang H, Diez-Sampedro A, You SA, et al. 2005. Calcium-sensitive potassium channelopathy in human epilepsy and paroxysmal movement disorder. *Nature genetics* 37: 733-8
19. Berkefeld H, Fakler B. 2013. Ligand-gating by Ca^{2+} is rate limiting for physiological operation of BK_{Ca} channels. *J Neurosci* 33: 7358-67
20. Park SM, Roache CE, Iffland PH, 2nd, Moldenhauer HJ, Matychak KK, et al. 2022. BK channel properties correlate with neurobehavioral severity in three *KCNMA1*-linked channelopathy mouse models. *Elife* 11: e77953
21. Buckley C, Williams J, Munteanu T, King M, Park SM, et al. 2020. Status Dystonicus, Oculogyric Crisis and Paroxysmal Dyskinesia in a 25 Year-Old Woman with a Novel KCNMA1 Variant, K457E. *Tremor Other Hyperkinet Mov* 10: 49
22. Yesil G, Aralasmak A, Akyuz E, Icagasioglu D, Uygur Sahin T, Bayram Y. 2018. Expanding the Phenotype of Homozygous *KCNMA1* Mutations; Dyskinesia, Epilepsy, Intellectual Disability, Cerebellar and Corticospinal Tract Atrophy. *Balkan Med J* 35: 336-39
23. Tian WT, Huang XJ, Mao X, Liu Q, Liu XL, et al. 2018. Proline-rich transmembrane protein 2-negative paroxysmal kinesigenic dyskinesia: Clinical and genetic analyses of 163 patients. *Mov Disord* 33: 459-67
24. Li X, Poschmann S, Chen Q, Fazeli W, Oundjian NJ, et al. 2018. De novo BK channel variant causes epilepsy by affecting voltage gating but not Ca^{2+} sensitivity. *Eur J Hum Genet* 26: 220-29
25. Zhang G, Gibson RA, McDonald M, Liang P, Kang PW, et al. 2020. A Gain-of-Function Mutation in *KCNMA1* Causes Dystonia Spells Controlled With Stimulant Therapy. *Mov Disord* 35: 1868-73
26. Tabarki B, AlMajhad N, AlHashem A, Shaheen R, Alkuraya FS. 2016. Homozygous *KCNMA1* mutation as a cause of cerebellar atrophy, developmental delay and seizures. *Hum Genet* 135: 1295-98
27. Al-Attas AA, Aldayel AY, Eskandrani AM, Biary N. 2022. KCNMA1-related refractory status epilepticus responding to vagal nerve stimulation: Case report and literature review. *Neurosciences* 27: 275-78
28. Zhang ZB, Tian MQ, Gao K, Jiang YW, Wu Y. 2015. De novo *KCNMA1* mutations in children with early-onset paroxysmal dyskinesia and developmental delay. *Mov Disord* 30: 1290-2
29. Keros S, Heim J, Hakami W, Zohar-Dayan E, Ben-Zeev B, et al. 2022. Lisdexamfetamine therapy in paroxysmal non-kinesigenic dyskinesia associated with the *KCNMA1*-N999S variant. *Movement Disorders Clinical Practice* 9: 229–35
30. de Gusmao CM. 2022. Teaching Video NeuroImage: Dystonic Cataplexy in KCNMA1 Paroxysmal Movement Disorder. *Neurology* 99:1010-1011
31. Heim J, Vemuri A, Lewis S, Guida B, Troester M, et al. 2020. Cataplexy in Patients Harboring the *KCNMA1* p.N999S Mutation. *Movement Disorders Clinical Practice* 7: 861-62

32. Wang J, Yu S, Zhang Q, Chen Y, Bao X, Wu X. 2017. *KCNMA1* mutation in children with paroxysmal dyskinesia and epilepsy: Case report and literature review. *Translational Science of Rare Diseases* 2: 8
33. Wu H, Li H, Bai T, Han L, Ou J, et al. 2020. Phenotype-to-genotype approach reveals head-circumference-associated genes in an autism spectrum disorder cohort. *Clin Genet* 97: 338-46

Downloaded from www.annualreviews.org. Guest (guest) IP: 173.79.247.107 On: Thu, 01 Oct 2026 23:36:35
